# Supplementary material for: Regulation of coordinated muscular relaxation in Drosophila larvae by a pattern-regulating intersegmental circuit
Source: Nat Commun. 2021 May 19;12:2943. doi: 10.1038/s41467-021-23273-y (PMC8134441; doi:10.1038/s41467-021-23273-y)
Supplement: Supplementary file 1 — Supplementary Information [file 41467_2021_23273_MOESM1_ESM.pdf]

Supplementary Figures

Regulation of coordinated muscular relaxation in *Drosophila* larvae by a pattern-regulating intersegmental circuit

Hiramoto *et al*.

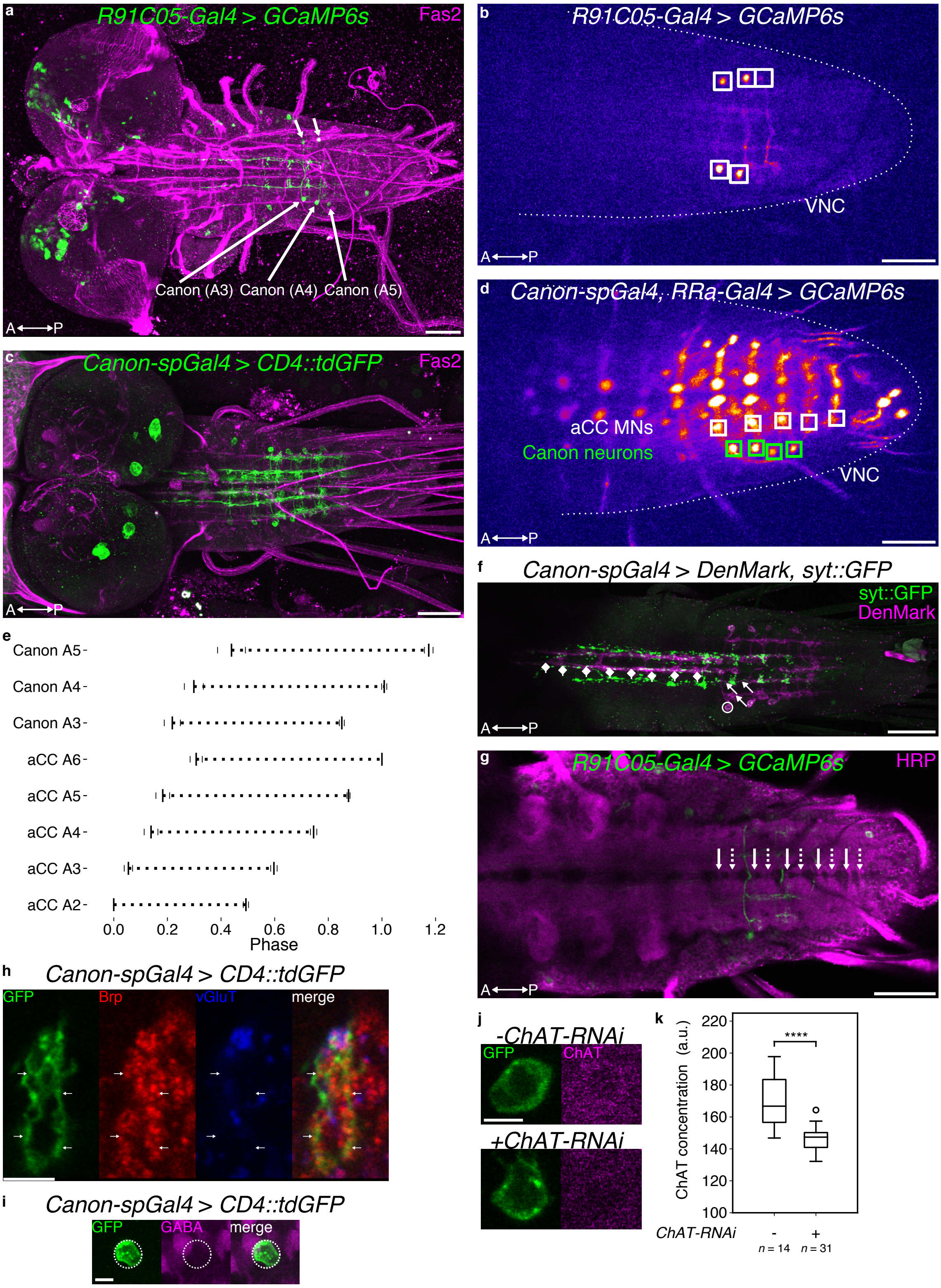

Supplementary Figure 1. Canon neurotransmitter phenotype and activity timing.

(a) Expression pattern of GCaMP6s driven by *R91C05-Gal4*. (b) Calcium imaging of Canon neurons. Regions of interest (ROIs) used for the analyses shown in Fig. 1b are indicated by squares. (c) Expression pattern of GFP driven by *Canon-spGal4*. (d) Calcium imaging of Canon neurons and aCC MNs. ROIs used for the analyses shown in Fig. 1c, d and Supplementary Fig. 1e are indicated by squares. (e) Plot of the activity timing of A2-A6 aCC MNs and A3-A5 Canon neurons. Normalized time duration from the onset to the peak of calcium signals in each neuron. Activity onset in A2 aCC is set as time 0.0 and peak time in A6 aCC is set as time 1.0. $n = 26$ waves from three larvae. The error bars represent standard errors of the mean. (f) Stacked focal planes showing the expression of postsynaptic (DenMark, magenta) and presynaptic (Syt-GFP, green) markers. Arrows indicate dendrites of Canon neurons. A circle indicates the cell body of a Canon neuron. Lines with square heads indicate dendrites and axons of Wave neurons. (g) Canon neurons (green) extend their axons along the anterior commissures. Anti-HRP (magenta) was used to identify anterior (solid arrows) and posterior (dotted arrows) commissures. (h) Presynaptic sites (Brp, red) of Canon neurons (GFP, green) do not overlap with vGluT concentration (vGluT, blue). (i) The cell body of a Canon neuron (GFP, green) is negative for GABA (magenta). (j) Canon neurons are immunoreactive to ChAT antibodies (top) and the signal is diminished by ChAT-RNAi (bottom). (k) Quantification of immunoreactivity to ChAT antibody using immunohistochemistry. n = 14 and 31 cells from three control and six experimental animals, respectively. Center line, median; box limits, upper and lower quartiles; whiskers, maximum and minimum between 1.5×interquartile range; points, outliers. ****$p<0.0001$. $p=7.66\times10^{-6}$, the two-sided Mann-Whitney U test. White dotted lines in (b, d) indicate boundaries of VNC. The images (a-d, f-i) are representative data of two (a), two (b), two (c), three (d), one (f), two (g), two (h), and two (i) experiments, respectively.

Scale bars, 50 µm (a-d, f, and g), and 5 µm (h-j).

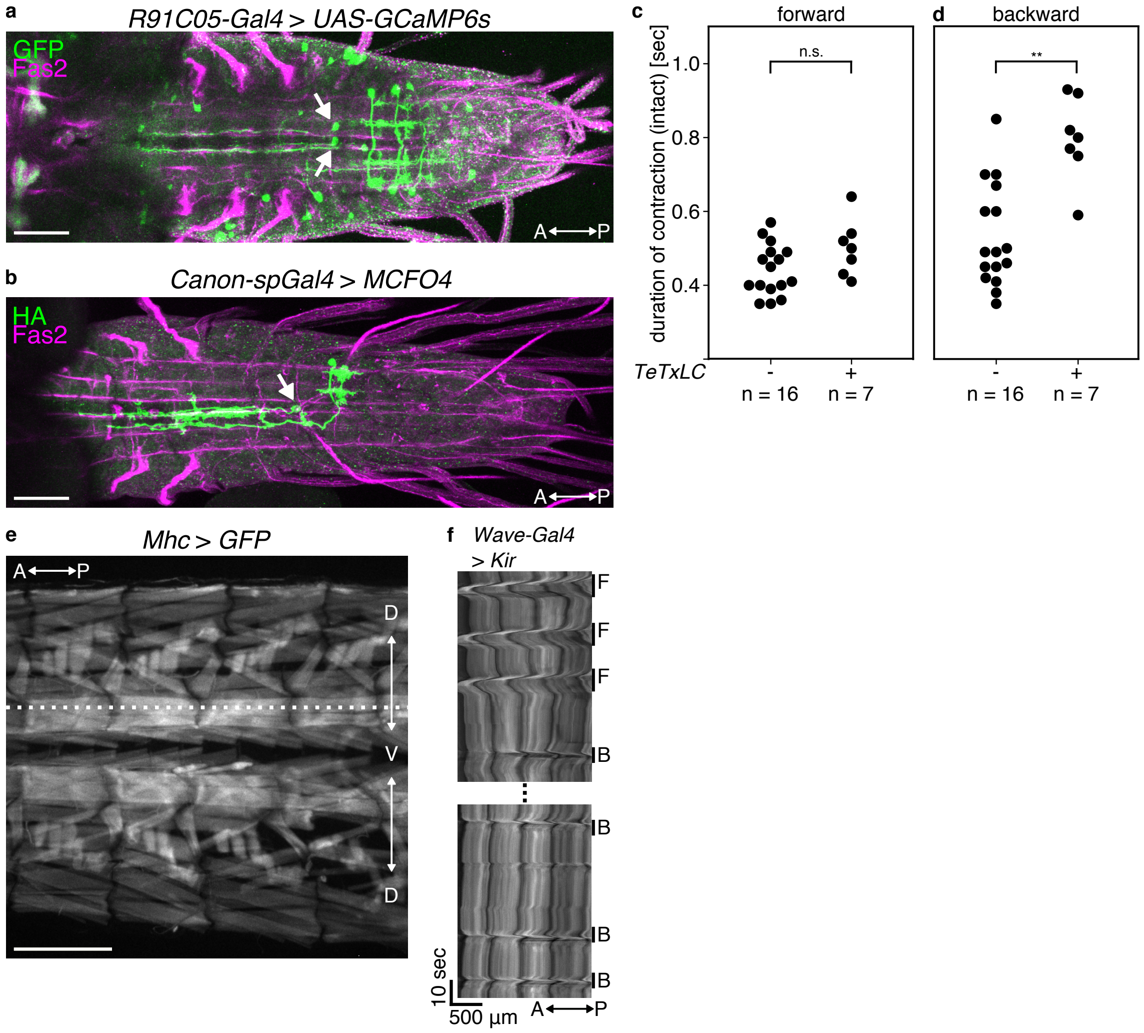

Supplementary Figure 2. Wave neurons play no roles in muscular relaxation.

(a, b) Anterior Wave neurons (arrows) targeted by *R91C05-Gal4* (a) and *Canon-spGal4* (b). The images are representative data of two (a) and 12 (b) experiments, respectively. (c, d) Comparison of duration of A2 contraction during forward (c) and backward (d) locomotion in intact larvae. n = 16 and 7 contractions in eight control and four experimental animals. **p<0.01, n.s.; not significant: p>0.05. $p=2.34\times10^{-3}$, the two-sided Mann-Whitney U test. (e, f) Normal peristalses in a dissected *MB120B-spGal4 > UAS-Kir* larva, in which Wave neurons were specifically inactivated. (e) An image of the body-wall muscles visualized by expression of *Mhc*GFP, focusing on ventral muscles in A2-A3

segments. The image is a representative datum of one experiment. (f) Kymograph generated from the white dotted line in (e) shows smooth muscular movement in both forward (F) and backward (B) peristalses. *n* = 11 forward and 7 backward waves from one larva. All peristalses examined were normal. Scale bars, 50 µm (a, b) and, 500 µm (e, f).

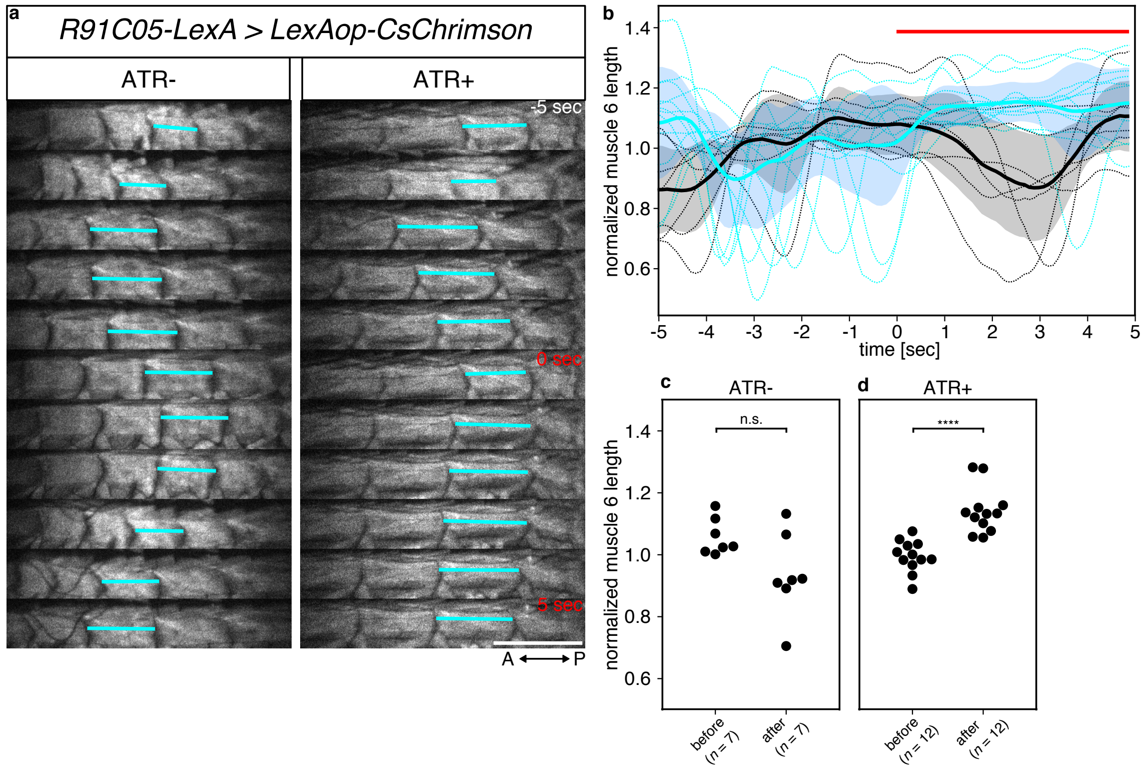

Supplementary Figure 3. Activation of Canon neurons induces muscular relaxation.

(a) Time-lapse images of body-wall muscles in control (left, ATR-) and experimental (right, ATR+) dissected larvae under optogenetic perturbation during sequential peristalses. All muscles in the experimental larvae relaxed immediately upon photostimulation, while the control larvae continued peristalsis. Cyan lines represent muscle 6 in A3 segment. 0 sec indicates the onset of light stimulation. (b) Muscular length normalized to the average length of muscle before stimulation. Cyan and black solid lines indicate the mean of experimental and control (ATR-) groups, respectively. The error bands (blue and gray shade) represent standard deviation. Cyan (experimental) and black (control) dotted lines indicate results of individual animals. Red bar shows the duration of photostimulation. (c, d) Quantification of normalized muscle 6 length before and after light stimulation in control (ATR-) and experimental groups, respectively.

Averaged muscle 6 length during the three-second period just before (before) and 1-4 second after (after) the onset of the stimulation. $n = 7$ and 12 peristalses in three control and three experimental larvae, respectively. ****$p<0.0001$, n.s.; not significant: $p>0.05$. $p=6.01\times10^{-5}$, the two-sided Mann-Whitney U test. Scale bar, 500 µm.

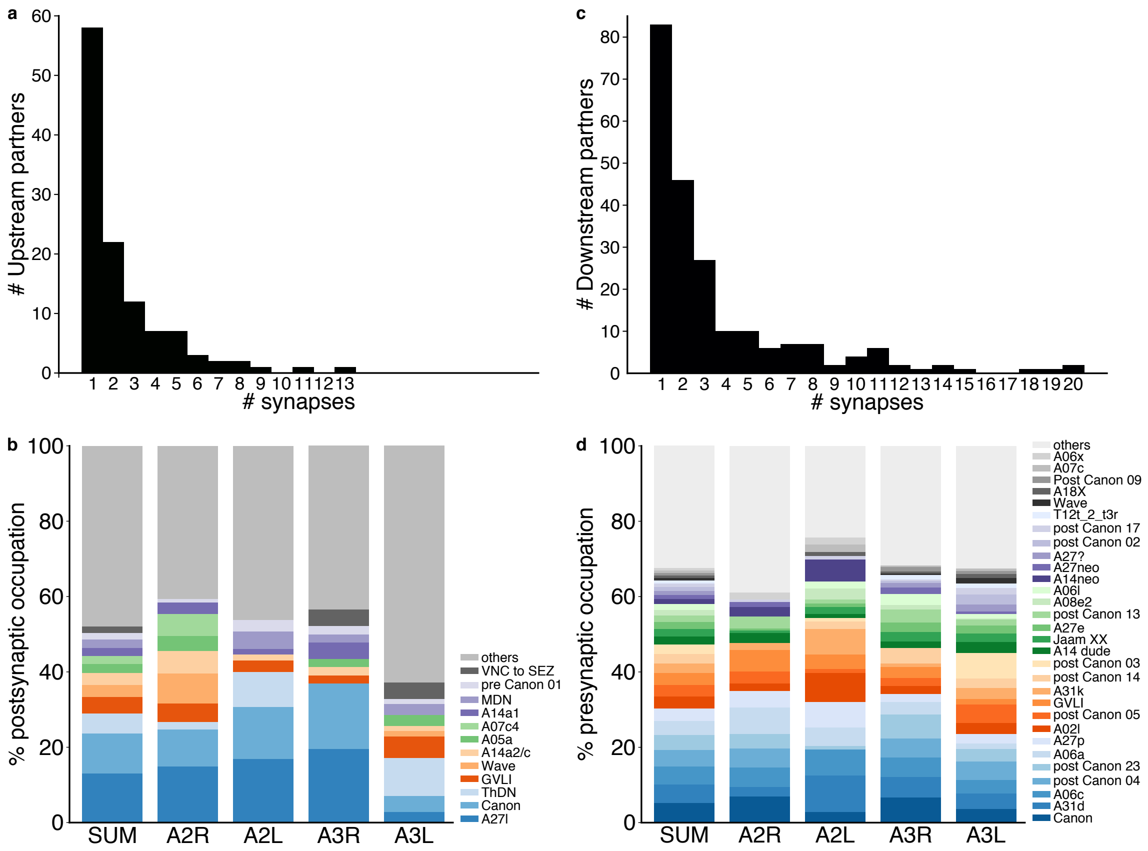

Supplementary Figure 4. Pre- and post-synaptic partners of Canon neurons.

(a, c) Distribution plots of presynaptic (i.e. upstream) (a) and post-synaptic (i.e. downstream) (c) partners of Canon neurons in A2. Transverse axes indicate the number of upstream or downstream neurons of Canon neurons. (b, d) Synaptic occupation by identified upstream (b) and downstream (d) partners of bilateral pairs of Canon neurons in A2 and A3. Neurons that are not shared or group of neurons that have a total of no more than four synapses are classified as “others”.

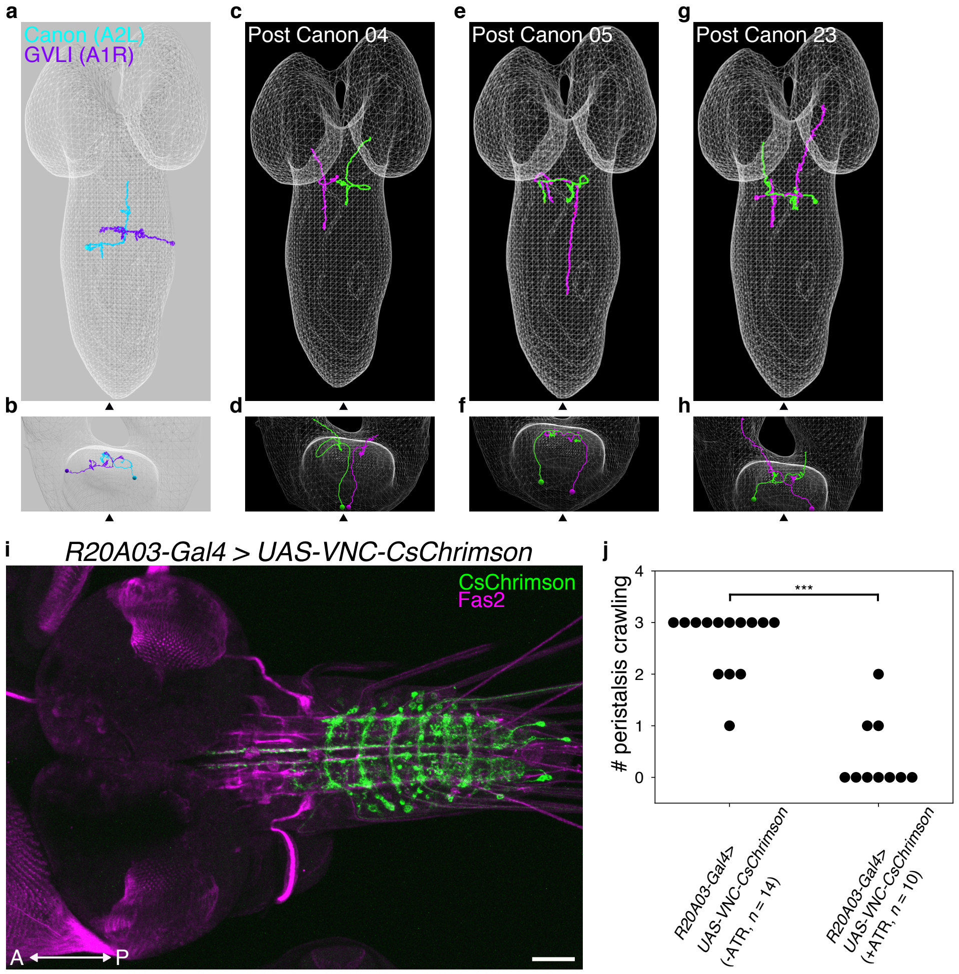

Supplementary Figure 5. Characterization of post-synaptic partners of Canon neurons.

(a-h) EM reconstructions of GVLI (a, b) and intersegmental neurons (c-h), downstream of Canon neurons. Dorsal (a, c, e, g) and anterior (b, d, f, h) view are shown. (i) Expression of CsChrimson driven by *R20A03-Gal4>UAS-VNC-CsChrimson*. (j) Quantification of the number of forward peristalses during the first 2 seconds of optogenetic stimulation of *R20A03-Gal4, UAS-VNC-CsChrimson* larvae. n = 14 and 10 peristaltic crawling in seven control (ATR-) and five experimental larvae. A majority of larvae in the experimental group stopped upon light stimulation, while control larvae were unaffected. ***$p<0.001$. $p=3.47\times10^{-4}$, the two-sided Fisher exact test. Scale bar, 50 µm.

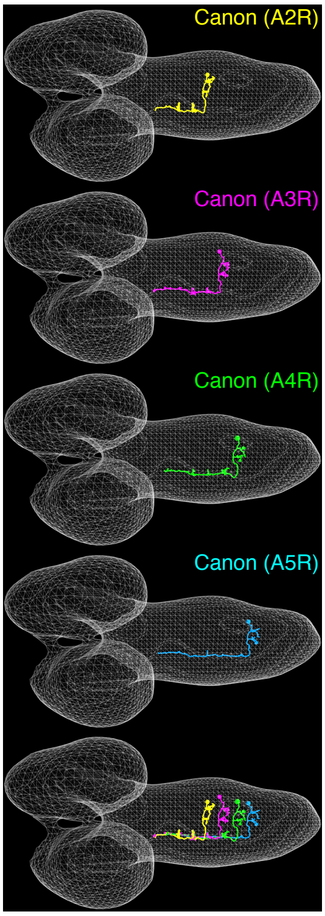

b

Pre \ Post	A2R	A3R	A4R	A5R
A2R	0	4.3	10	2.0
A3R	4.0	0	10	6.0
A4R	3.0	11	0	4.0
A5R	1.0	2.2	7.7	0

Supplementary Figure 6. Reconstruction of Canon in each neuromere.

(a) EM reconstruction of right-side Canon neurons located in segments A2-A5. (b) A table of percentage of postsynapse occupation between Canon neurons. Canon neurons form synapses with each other bidirectionally.

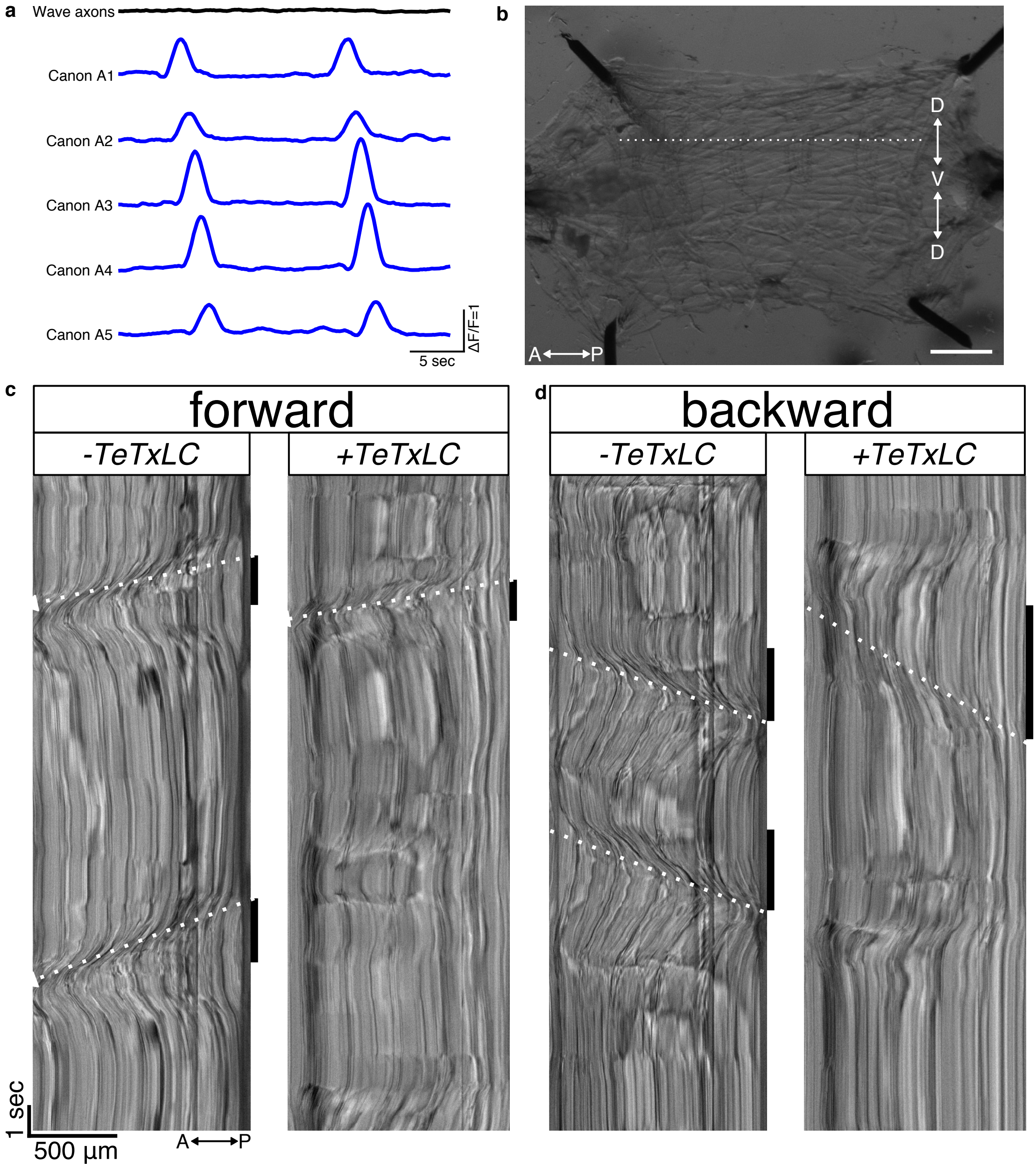

Supplementary Figure 7. *Canon-spGal4 > TeTxLC* semi-intact preparations generate complete backward peristalses.

(a) Simultaneous calcium imaging of Wave (black) and Canon (blue) neurons in isolated CNS. Note that during the propagation of Canon activation, Wave neurons show no activity. Thus, Wave neurons are unlikely to have roles in the activity propagation of Canon neurons. (b) An image of a dissected larva used for the analysis. The dotted white line indicates the location used to make the kymographs shown in (c, d). The image is a

representative datum of seven experiments. (c, d) Kymographs showing the propagation of muscle contraction (arrows with dotted lines) during forward (c) and backward (d) peristalses in control (-*TeTxLC*) and *Canon > TeTxLC* (+*TeTxLC*) larvae. Note that all backward peristalses propagate all the way through in the *Canon-spGal4 > TeTxLC* larva as in the control, although the propagation is slower as observed in the *Canon-spGal4 > Kir* larvae. n = 8 backward peristalses in two control larvae and 9 backward peristalses in five experimental larvae. This indicates that propagation of excitatory drive is intact in *Canon-spGal4 > TeTxLC* larvae. Scale bars, 500 µm (b, c).

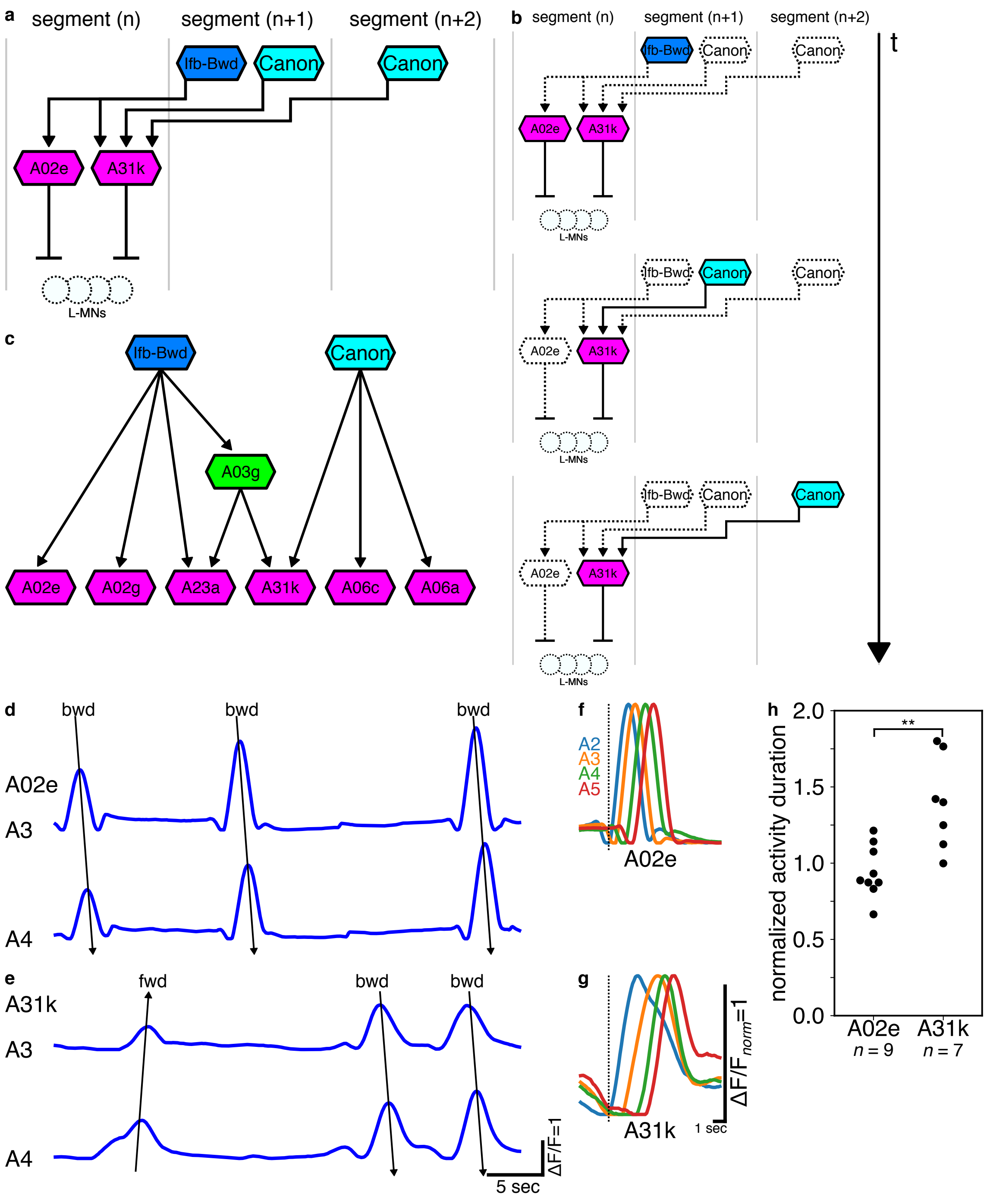

Supplementary Figure 8. Sequential activation of inhibitory premotor neurons by Ifb-Bwd and Canon neurons.

(a) Co-innervation of the A31k premotor neuron by Ifb-Bwd and Canon neurons. A circuit diagram showing the connections between inhibitory premotor neurons, A31k and A02e, and higher-order Ifb-Bwd and Canon neurons. A02e in segment (n) receives inputs from

Ifb-Bwd in segment (n+1) but not from Canon, whereas A31k in segment (n) receives inputs from Ifb-Bwd in segment (n+1) and Canon in segment (n+1) and (n+2). Arrows with filled heads indicate cholinergic synapses while bar heads indicate inhibitory outputs. (b) A scheme showing sequential and long-lasting activation of A31k by Ifb-Bwd and Cannon neurons. (c) A wiring diagram of the inhibitory premotor neurons downstream of Ifb-Bwd and Canon neurons. (d-h) Longer activation of A31k compared to A02e. Reanalysis of data obtained in a previous study[1] (d, e) Plots of the normalized GCaMP6f calcium signal of A02e (d) and A31k (e) during fictive locomotion. (f, g) Normalized GCaMP6f signals of A02e (f) and A31k (g) located in A2-5 neuromeres during fictive backward locomotion. In (d-g), wave propagation revealed by RGECO1 signals in A02e neurons was used to determine the motor phase (see Methods). (h) Quantification of normalized activity duration of A02e and A31k in the A3 neuromere. Note that activity duration of A31k is significantly longer than A02e. n = 9 waves in five larvae for A02e and 7 waves in three larvae for A31k. **p<0.01. $p<5.84\times10^{-3}$. The two-sided Mann-Whitney U test.

Supplementary References

1. Kohsaka, H. *et al.* Regulation of forward and backward locomotion through intersegmental feedback circuits in *Drosophila* larvae. *Nat. Commun.* **10**, 1–11 (2019).
