# Supplementary material for: Regulation of coordinated muscular relaxation in Drosophila larvae by a pattern-regulating intersegmental circuit
Source: Nat Commun. 2021 May 19;12:2943. doi: 10.1038/s41467-021-23273-y (PMC8134441; doi:10.1038/s41467-021-23273-y)
Supplement: Supplementary file 3 — Description of Additional Supplementary Files [file 41467_2021_23273_MOESM3_ESM.docx]

**Supplementary Movie 1. Canon neurons wave like activity with backward direction.**

Calcium imaging of an isolated larval CNS expressing GCaMP6s under *R91C05-Gal4*. The anterior direction of the CNS is the left side of this movie. The speed of this video is three times the original data. The movie shows representative fictive locomotion during this recording.

**Supplementary Movie 2. Canon neurons show wave like activity during fictive backward locomotion.**

Calcium imaging of an isolated larval CNS expressing GCaMP6s in Canon neurons and aCC MNs. The anterior direction of the CNS is the left side of this movie. The appearance of white arrows indicates the onsets of increment of GCaMP6s fluorescent intensity in aCC MNs and their locations indicate the locations of dendrites of aCC MNs. The appearance of green arrows indicates the onsets of increment of GCaMP6s fluorescent intensity in Canon neurons and their locations indicate the locations of cell bodies of Canon neurons. The speed of this video is the same as the original data and a 20X water immersion objective lens was used in this recording. The movie shows representative fictive locomotion during this recording.

**Supplementary Movie 3. A larva stops locomotion immediately when Canon neurons are optogenetically activated.**

Optogenetical activation of Canon neurons specifically with CsChrimson during sequential forward locomotion. The speed of this video is the same as the original data.

The movie shows representative trials of 10 experiments with control (ATR-) animals and 10 experiments with experimental animals. The anterior direction of the CNS is the left side of this movie. The movie shows representative fictive backward locomotion during this recording.

**Supplementary Movie 4. Slower muscular relaxation during backward peristalsis of a *Canon-spGal4 > UAS-Kir* animal.**

Backward peristalsis of dissected larvae expressing GFP on their muscles and expressing *Canon-spGal4 > UAS-Kir* in the experimental animal. The speed of this video is three times the original data. The anterior direction of the CNS is the left side of this movie. The movie shows representative fictive backward locomotion during this recording.

**Supplementary Movie 5. Termination of Canon activity propagation in a *Canon-spGal4 > UAS-TeTxLC* animal.**

Calcium imaging of Canon neurons under chemical synapses inhibition via expression of TeTxLC. The speed of this video is three times of the original data. The anterior direction of the CNS is the left side of this movie. The movie shows representative fictive backward locomotion during this recording.
