# Supplementary material for: Regulation of coordinated muscular relaxation in Drosophila larvae by a pattern-regulating intersegmental circuit
Source: Nat Commun. 2021 May 19;12:2943. doi: 10.1038/s41467-021-23273-y (PMC8134441; doi:10.1038/s41467-021-23273-y)
Supplement: Supplementary file 9 — Reporting Summary [file 41467_2021_23273_MOESM9_ESM.pdf]

nature research

Corresponding author(s): Akinao Nose

Last updated by author(s): Apr 20, 2021

Reporting Summary

Nature Research wishes to improve the reproducibility of the work that we publish. This form provides structure for consistency and transparency in reporting. For further information on Nature Research policies, see our Editorial Policies and the Editorial Policy Checklist.

Statistics

For all statistical analyses, confirm that the following items are present in the figure legend, table legend, main text, or Methods section.

n/a	Confirmed	
☐	☒	The exact sample size (n) for each experimental group/condition, given as a discrete number and unit of measurement
☐	☒	A statement on whether measurements were taken from distinct samples or whether the same sample was measured repeatedly
☐	☒	The statistical test(s) used AND whether they are one- or two-sided<br>*Only common tests should be described solely by name; describe more complex techniques in the Methods section.*
☒	☐	A description of all covariates tested
☐	☒	A description of any assumptions or corrections, such as tests of normality and adjustment for multiple comparisons
☐	☒	A full description of the statistical parameters including central tendency (e.g. means) or other basic estimates (e.g. regression coefficient) AND variation (e.g. standard deviation) or associated estimates of uncertainty (e.g. confidence intervals)
☐	☒	For null hypothesis testing, the test statistic (e.g. F, t, r) with confidence intervals, effect sizes, degrees of freedom and P value noted<br>*Give P values as exact values whenever suitable.*
☒	☐	For Bayesian analysis, information on the choice of priors and Markov chain Monte Carlo settings
☒	☐	For hierarchical and complex designs, identification of the appropriate level for tests and full reporting of outcomes
☒	☐	Estimates of effect sizes (e.g. Cohen's d, Pearson's r), indicating how they were calculated

Our web collection on statistics for biologists contains articles on many of the points above.

Software and code

Policy information about availability of computer code

Data collection	Fluoview Version.4.2 (Olympus), iQ 3.0(ANDOR TECHNOLOGY), VFS-42 Version 4.01 (Chori imaging), CATMAID Version 2020.02.15-989-g90b1a5e76
Data analysis	Python 3.6, imageJ Version: 2.0.0-rc-68/1.52e

For manuscripts utilizing custom algorithms or software that are central to the research but not yet described in published literature, software must be made available to editors and reviewers. We strongly encourage code deposition in a community repository (e.g. GitHub). See the Nature Research guidelines for submitting code & software for further information.

Data

Policy information about availability of data

All manuscripts must include a data availability statement. This statement should provide the following information, where applicable:

- Accession codes, unique identifiers, or web links for publicly available datasets
- A list of figures that have associated raw data
- A description of any restrictions on data availability

All data supporting the findings of this study are provided within the paper and its supplementary information. Source data are provided with this paper.

Field-specific reporting

Life sciences study design

All studies must disclose on these points even when the disclosure is negative.

Sample size	We chose sample size by referring to our previous study (Kohsaka et al., 2019) where the difference in larval behavior was successfully quantified.
Data exclusions	No data ere excluded.
Replication	We conducted each experiment for multiple days and the data of each experiments group were merged into single data.
Randomization	We randomly picked up larvae from a large population of fly larvae (more than 100) of each genotype in all experiments.
Blinding	We were not blinded to group allocation. Since each experimental group consists of a distinct genotype, we did not allocate larvae of a certain genotype into multiple groups in this study. Accordingly, we do not think we need to be blinded in this step.

Reporting for specific materials, systems and methods

We require information from authors about some types of materials, experimental systems and methods used in many studies. Here, indicate whether each material, system or method listed is relevant to your study. If you are not sure if a list item applies to your research, read the appropriate section before selecting a response.

Materials & experimental systems

n/a	Involved in the study	
☐	☒	Antibodies
☒	☐	Eukaryotic cell lines
☒	☐	Palaeontology and archaeology
☐	☒	Animals and other organisms
☒	☐	Human research participants
☒	☐	Clinical data
☒	☐	Dual use research of concern

Methods

n/a	Involved in the study	
☒	☐	ChIP-seq
☒	☐	Flow cytometry
☒	☐	MRI-based neuroimaging

Antibodies

Antibodies used

rabbit anti-GFP (Af2020, Frontier Institute; 1:1000; RRID: AB 2571573), guinea pig anti-GFP (Af1180, Frontier Institute; 1:1000; RRID: AB 2571575), mouse anti-FasII (1D4, Hybridoma Bank (University of Iowa); 1:10; RRID: AB 528235), rabbit anti-HA (C29F4, Cell Signaling Technology; 1:300; RRID: AB 1549585), rat anti-FLAG (NBP1-06712, Novus Biologicals; 1:200; RRID: AB 1625981), mouse anti-ChAT (4B1, Hybridoma Bank (University of Iowa); 1:50; RRID: AB 528122), rabbit anti-vGluT (Gift from Dr. Hermann Aberle; vGluT, Max-Planck-Institute for Developmental Biology; 1:1000; RRID: AB 2315544), mouse anti-Brunchpilot (Brp) (nc82, Hybridoma Bank (University of Iowa); 1:50; RRID: AB 2314866), rabbit anti-DsRed (632496, Clontech; 1:500; RRID: AB 0015246), goat Alexa Fluor 488 anti-rabbit (A11034, Thermo Fisher Scientific; 1:300; RRID AB 2576217), goat Cy3 anti-rabbit (A10520, Thermo Fisher Scientific; 1:300; RRID: AB 10563288), goat Cy5 anti-rabbit (A10523, Thermo Fisher Scientific; 1:300; RRID: AB 2534032), goat Alexa Fluor 555 anti-mouse (A21424, Thermo Fisher Scientific; 1:300; RRID: AB 141780), goat Cy5 anti-mouse (A10524, Thermo Fisher Scientific; 1:300; RRID: AB 2534033), goat Alexa Fluor 633 anti-rat (A21094, Thermo Fisher Scientific; 1:300; RRID: AB 141553), goat Alexa Fluor 488 anti-guinea pig (A11073, Thermo Fisher Scientific; 1:300; RRID: AB 2534117), goat Alexa Fluor 647 anti-Horseradish Peroxidase (HRP) (123-605-021, Jackson ImmunoResearch; 1:200; RRID: AB 2338967)

Validation

rabbit anti-GFP (Af2020, Frontier Institute)
https://www.frontier-institute.com/wp/wp-content/uploads/pdf/GFP.pdf
Specificity : Mouse (others not tested). This selectively stains particular types of cells in GFP-transgenic mice, but not wild-type mice.
Kohsaka, H. et al. Regulation of forward and backward locomotion through intersegmental feedback circuits in Drosophila larvae. Nat. Commun. 10, 1–11 (2019).

guinea pig anti-GFP (Af1180, Frontier Institute)
https://www.frontier-institute.com/wp/wp-content/uploads/pdf/GFP.pdf
Specificity : Mouse (others not tested). This selectively stains particular types of cells in GFP-transgenic mice, but not wild-type mice.
Kohsaka, H. et al. Regulation of forward and backward locomotion through intersegmental feedback circuits in Drosophila larvae. Nat. Commun. 10, 1–11 (2019).

mouse anti-FasII (1D4, Hybridoma Bank (University of Iowa))
https://dshb.biology.uiowa.edu/1D4-anti-Fascicli-II
Positive Tested Species Reactivity: Drosophila
Van Vactor, David, et al. "Genes that control neuromuscular specificity in Drosophila." Cell 73.6 (1993): 1137-1153.

rabbit anti-HA (C29F4, Cell Signaling Technology)

https://www.cellsignal.com/products/primary-antibodies/ha-tag-c29f4-rabbit-mab/3724
Specificity / Sensitivity: HA-Tag (C29F4) Rabbit mAb detects exogenously expressed proteins containing the HA epitope tag. The antibody may cross-react with a protein of unknown origin ~100kDa.
Species Reactivity: All Species Expected
Takagi, S. et al. Divergent Connectivity of Homologous Command-like Neurons Mediates Segment-Specific Touch Responses in Drosophila. Neuron 96, 1373-1387.e6 (2017).

rat anti-FLAG (NBP1-06712, Novus Biologicals)
https://www.novusbio.com/products/dykddddk-epitope-tag-antibody-l5_nbp1-06712
Reactivity Notes: Tag specific. Reactivity reported in multiple pieces of scientific literature. (PMID: 25896325), (PMID: 28651121).
Nern, A., Pfeiffer, B. D. & Rubin, G. M. Optimized tools for multicolor stochastic labeling reveal diverse stereotyped cell arrangements in the fly visual system. Proc. Natl. Acad. Sci. U. S. A. 112, E2967–E2976 (2015).

mouse anti-ChAT (4B1, Hybridoma Bank (University of Iowa))
https://dshb.biology.uiowa.edu/ChAT4B1
Positive Tested Species Reactivity: Drosophila, Manduca sexta
Takagawa, Kiyoshi, and Paul Salvaterra. "Analysis of choline acetyltransferase protein in temperature sensitive mutant flies using newly generated monoclonal antibody." Neuroscience research 24.3 (1996): 237-243.

rabbit anti-vGluT (vGluT, Max-Planck-Institute for Developmental Biology)
https://antibodyregistry.org/search.php?q=AB_2315544%3c/result%3e
Kohsaka, H. et al. Regulation of forward and backward locomotion through intersegmental feedback circuits in Drosophila larvae. Nat. Commun. 10, 1–11 (2019).

mouse anti-Brunchpilot (Brp) (nc82, Hybridoma Bank (University of Iowa))
https://dshb.biology.uiowa.edu/nc82
Positive Tested Species Reactivity: Drosophila, Mosquito
Ohyama, T. et al. A multilevel multimodal circuit enhances action selection in Drosophila. Nature 520, 633–639 (2015).

rabbit anti-DsRed (632496, Clontech)
https://www.takarabio.com/documents/Certificate%20of%20Analysis/632496/632496-101717.pdf
Description: The Living Colors DsRed Polyclonal Antibody has been raised against DsRed-Express, a variant of Discosoma sp. red fluorescent protein. This antibody recognizes DsRed-Express, DsRed-Express2, mCherry, DsRed2, E2-Crimson, tdTomato, mStrawberry, and mBanana, and both N- and C-terminal fusion proteins containing these fluorescent proteins in mammalian cell lysates.
Kohsaka, H. et al. Regulation of forward and backward locomotion through intersegmental feedback circuits in Drosophila larvae. Nat. Commun. 10, 1–11 (2019).

Animals and other organisms

Policy information about studies involving animals; ARRIVE guidelines recommended for reporting animal research

Laboratory animals

Drosophila melanogaster
Third-instar larvae collected without selection based on sex were used for all functional and histological experiments.
y1 w1118 (Bloomington Drosophila Stock Center (BDSC), #6598)
R91C05-Gal4 (BDSC, #40578)
R26A08-Gal4 (BDSC, #49153)
R20A03-Gal4 (BDSC, #48871)
RRa-Gal4 (gift from Dr. Miki Fujioka)
UAS-GCaMP6s (BDSC, #42746)
UAS-CD4::GCaMP6f
UAS-TeTxLC (BDSC, #28838)
UAS-Kir2.1::EGFP (BDSC, #6596)
UAS-CD4::tdGFP (BDSC, #35836)
UAS-DenMark, UAS-syt::GFP/CyO; D/TM6C (UAS-TLN-21) (gift from Dr. Bassem A. Hassan)
MCFO4 (BDSC, #64087)
R91C05-LexA (BDSC, #61629)
LexAop-CsChrimson::mVenus, (BDSC, #55136)
UAS-VNC-CsChrimson (20xUAS>dsFRT>CsChrimson::mVenus in attP18; tsh-LexA, pJFRC79-8xLexAop2-FlpL in attP40, this study)
MhcGFP (gift from Dr. Cynthia L. Hughes)
R91C05-Gal4.AD (BDSC, #70979)
VT019059-Gal4.DBD (BDSC, #71731)
MB120B-spGal4
Cha3.3kbp-Gal80 (gift from Dr. Toshihiro Kitamoto)

Wild animals

This study did not involve wild animals.

Field-collected samples

This study did not involve samples collected from the field.

Ethics oversight

No ethical approval was required in the study using Drosophila melanogaster.

Note that full information on the approval of the study protocol must also be provided in the manuscript.
